# Recycled Aggregate: A Solution to Sustainable Concrete

**DOI:** 10.3390/ma18122706

**Published:** 2025-06-09

**Authors:** Jitao Bai, Chenxi Ge, Jiahe Liang, Jie Xu

**Affiliations:** 1 Department of Civil Engineering, Tianjin University, Tianjin 300072, China; jitaobai_123@tju.edu.cn; 2 Key Laboratory of Coast Civil Structures and Safety of Ministry of Education, Tianjin University, Tianjin 300072, China; 3 School of Architecture, Tianjin University, Tianjin 300072, China; 18805559522@163.com; 4 Faculty of Architecture, KU Leuven, 9000 Gent, Belgium; liangjiahe117@gmail.com; 5 Key Laboratory of Earthquake Engineering Simulation and Seismic Resilience of China Earthquake Administration, Tianjin University, Tianjin 300072, China

**Keywords:** recycled aggregate, recycled aggregate concrete, material properties, test, modeling, improvement, application

## Abstract

Recycling construction and demolition (C&D) waste into recycled aggregate (RA) and recycled aggregate concrete (RAC) is conducive to natural resource conservation and industry decarbonization, which have been attracting much attention from the community. This paper aims to present a synthesis of recent scientific insights on RA and RAC by conducting a systematic review of the latest advances in their properties, test techniques, modeling, modification and improvement, as well as applications. Over 100 papers published in the past three years were examined, extracting enlightening information and recommendations for engineering. The review shows that consistent conclusions have been drawn about the physical properties in that RA can reduce the workability and the setting time of fresh RAC and increase the porosity of hardened RAC. Its impact on drying and autogenous shrinkage is governed by its size and the strength of the parent concrete. RA generally acts negatively on the durability and mechanical properties of concrete, but such effects remain controversial as many opposite observations have been reported. Apart from the commonly used multiscale test techniques, real-time monitoring also plays an important role in the investigation of deformation and fracture processes. Analytical models for RAC were usually modified from the existing models for NAC or established through regression analysis, while for numerical models, the distribution of attached mortar should be considered to improve their accuracy. Machine learning models are effective in predicting RAC properties. Modification of RA can be implemented by either removing or strengthening the attached mortar, while the modification of RAC is mainly achieved by improving its microstructure. Current exploration of RAC applications mainly focuses on the optimization of concrete design and mix procedures, structural components, as well as multifunctional construction materials, revealing the room for its further exploitation in the industry.

## 1. Introduction

Concrete is the most consumed material around the world by mass except for water [1]. Each year, about 30 billion tonnes of concrete are used worldwide [2], and the demand is still growing—much more steeply than that for other construction materials such as steel or timber [1]. Such tremendous consumption contributes heavily to the environmental issue of global warming. In fact, concrete has been identified as one of the biggest climate culprits [3], largely due to its significant carbon emissions. Manufacturing concrete is responsible for over 9% of the global anthropogenic carbon emissions [1]. In the United States, a survey on the office buildings constructed between 1946 and 2018 revealed that concrete contributes around 22% of the total embodied greenhouse gas emissions [4]. All these factors have driven the construction industry to be the biggest source of global emissions [5].

In addition to the aforementioned carbon footprint, large-scale production of concrete also causes the depletion of essential natural resources such as gravel and river sand and, once used in construction, brings overwhelming construction and demolition (C&D) waste to deal with. Statistics show that construction materials now account for half of the solid waste generated in the world, and in the US, concrete makes up 68% of the total C&D waste [4]. This considerable amount of waste is filling up landfills all over the world, burdening the local community both environmentally and economically [6].

Recycled aggregate (RA) has provided a sustainable solution to these problems. It not only finds a way for appropriate utilization of C&D waste, but also reduces the demand for natural resources while further lowering the cost of concrete [7,8]. Moreover, it shows great potential in concrete decarbonization. Studies found that over 14% of concrete-related carbon emissions come from aggregate, second only to cement among all the raw materials [9]. Replacing all the natural aggregate (NA) with coarse recycled aggregate can reduce lifecycle carbon emissions by at least 1–2% [10]. In a specific case, replacing all the NA in masonry blocks with RA reduced the global warming potential by 3.9% and the non-renewable energy consumption by 6.9% [11]. With proper treatment such as accelerated carbonation, the contribution from aggregate production to global warming potential can even achieve negative values in concrete with NA fully replaced by recycled concrete aggregate [12]. Indeed, RA is showing advantages over NA in terms of environmental impact, economy, and public perception [13].

**Figure 1:**
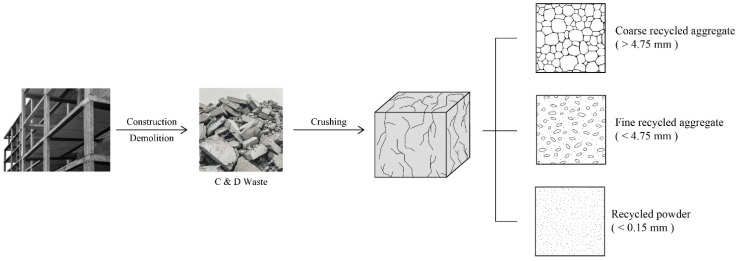
Production and different categories of recycled aggregate.

Most RA is produced from crushed waste concrete, i.e., recycled concrete aggregate (RCA). Sometimes, recycled brick aggregate (RBA) may also be generated from masonry structures. Based on the size distribution [14], RA can be classified into coarse recycled aggregate (CRA, >4.75 mm) and fine recycled aggregate (FRA, <4.75 mm), as illustrated in Figure 1. Even finer particles (<0.15 mm) can be obtained as well during the crushing of C&D waste, namely recycled powder (RP). To date, extensive research has been conducted on RA and recycled aggregate concrete (RAC), and indeed, it is not a newly raised topic. However, it cannot be denied that the research on RA and RAC is still active nowadays, with a mass of new knowledge contributing to this field each year. This paper makes an attempt to reflect the latest advances in RA and RAC research by giving a comprehensive review of the literature published in the past three years. Over 100 papers were systematically analyzed, aiming to provide the most up-to-date information for the application of RA and RAC in engineering.

The review is organized as follows. In Section 2, the properties of RAC, including the basic physical, mechanical, and durability properties, are critically reviewed. Test techniques typically involved in RAC research are briefly summarized in Section 3. In Section 4, the advances in both the analytical and numerical modeling of RAC are introduced. Regarding the generally poor performance of RAC, modification and improvement measures are discussed in Section 5, and the progress made in RAC applications is presented in Section 6. The conclusions are drawn in Section 7.

## 2. Material Properties

### 2.1. Basic Physical Properties

Consistent observations have been made on the physical properties of RA and RAC. Compared with NA, RA usually has lower density and higher water absorption due to the porous structure of the attached mortar. As a result, concrete produced with RA also exhibits higher void volume (porosity) and air content [15,16] characterized by lower ultrasonic pulse velocity (UPV) [17,18] as well as increased water and capillary absorption [15,19,20,21,22]. Specifically, pores > 100 nm in RA usually contribute to higher initial and saturation water absorption rates as well as stronger continuous water reversion ability, while those < 100 nm are mainly related to stronger continuous water absorption ability and higher residual water content in water reversion tests [23]. For fresh RAC, RA reduces the workability or slump [16,19], which is more pronounced for FRA [24] and RP [25] than for CRA. In addition, due to the high water absorption, FRA and RP also increase water consumption during concrete mixing [21] and, at the same time, shorten the setting time of fresh concrete [21,22]. In self-compacting concrete, RA was found to have a significant negative impact on the segregation resistance of concrete as well [26]. For hardened RAC, RA increases the drying shrinkage and surface cracks as well as the mass loss caused by water evaporation [16,17,27,28,29], but it also alleviates autogenous shrinkage [28,29]. However, the effect of RA on concrete shrinkage is affected by its size and the strength of the parent concrete from which it is produced. Smaller RA in size (in extreme cases, RP) and higher strength grade of the parent concrete can significantly reduce the drying shrinkage [22,25,29] but have little impact on autogenous shrinkage [29]. In addition to the impact on concrete shrinkage, RA leads to lower thermal conductivity as well [18,30,31]. Adopting two-stage mixing approach could have a positive effect on the workability and drying shrinkage of RAC [32].

The effects of RA on the basic physical properties of RAC are summarized in Table 1.

### 2.2. Mechanical Properties

**Figure 2:**
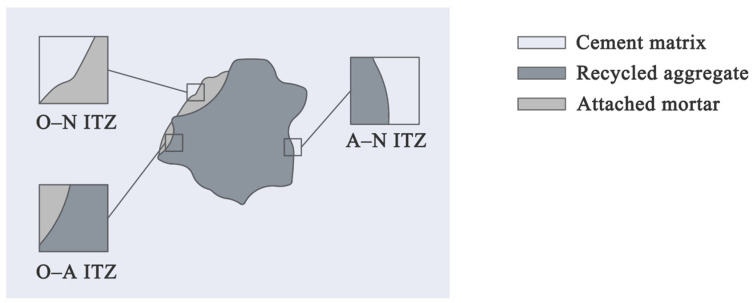
Interfacial transition zones in recycled aggregate concrete.

Generally, RA acts negatively on the mechanical properties of concrete [33], leading to decreased compressive strength [19,28], tensile strength [17], and elastic modulus [28]. Particularly, FRA has a more serious negative impact than CRA on the mechanical properties of RAC [18]. The strength degradation caused by RA is mainly attributed to the porous microstructure of the attached mortar, which not only has lower strength, but also attracts the migration of ions from the cement matrix and thereby reduces the hydration products [34]. This is partially evidenced by the more serious strength decline caused by FRA than CRA, as the content of the attached mortar tends to increase as the size of the RCA gets smaller [35]. Furthermore, RA usually forms weaker interfacial transition zones (ITZs) in concrete. Typically, the ITZs in RAC can be classified into three categories, that is, the interface between the attached old mortar on RA and the new cement matrix (O–N type), the interface between the bare aggregate of RA and the new cement matrix (A–N type), as well as the one between the wrapped aggregate in RA and its surrounding old mortar attached (O–A type), as shown in Figure 2. The difference between various types of ITZs mainly lies in the average elastic modulus and width (the width of the O–N-type ITZ is generally greater than that of the A–O type and the A–N type), while other properties such as the content of unhydrated cement as well as the average porosity show less difference [36]. Compared with the ITZs in natural aggregate concrete (NAC), those in RAC are more easily damaged under loads. However, ITZs are not always the weak part of RAC. A study on geopolymer RAC prepared with granulated blast furnace slag, fly ash, and recycled concrete powder observed that the A–N and O–N-type ITZs exhibited even higher elastic modulus than the cement matrix [37].

An interesting phenomenon is that great disparity, or even opposite conclusions, have been reported in the literature about how RA actually influences the strength of RAC. In [38], RA was found to be able to achieve a 14% higher compressive strength in RAC. That was attributed to the higher water absorption of RA caused by its high porosity, which reduces the effective water-to-cement ratio (W/C) and thereby leads to a denser microstructure in RAC. Such a strength-enhancing effect brought by RA is amplified when RAC is cured by CO_2_, as the abundant pores in RA facilitate CO_2_ diffusion in RAC to promote concrete carbonation. The compressive strength of RAC was found to follow a robust linear correlation with the porosity of RAC as well as the modulus and hardness of ITZs. This surprising strength improvement after RA incorporation is not an isolated case. Instead, many studies have observed a similar effect, particularly those conducted with FRA. For example, Rifa et al. [20] found that fine recycled concrete aggregate (FRCA) could improve the flexural and tensile strength as well as the abrasion resistance of concrete by 15%, 3.22%, and 30.1%, respectively, at different replacement rates and delay crack formation and propagation in hardened concrete. Based on the results, replacement rates of river sand by FRCA were recommended to be 15% and 60%, respectively, for concrete bearing tensile and flexural loads. An enhancement in the early compressive strength of concrete was witnessed as well in [21] with some specific amount of FRA. These unusual strength variations may be attributed to the differences in RA sources, testing standards, strength measurement methods, or RA moisture content, but more in-depth explanations have been given from the perspective of the hydration process. One view is that the high content of silica and alumina as well as CaO in FRA promotes secondary pozzolanic reactions in concrete [16] and leads to additional hydration products such as CaCO_3_, C–S–H gel, and vanadate, thereby significantly increasing the fracture energy of RAC [39]. Similarly, recycled concrete powder (RCP) can release Ca^2+^ during its secondary hydration, which promotes the formation of high-strength and highly stable C–S–H and C–A–S–H gels and thereby enhances the mechanical strength of concrete [40]. However, these explanations are only applicable to FRA or RP, as CRA seems to have no obvious impact on cement hydration [41].

The strength properties and failure mechanisms of RAC were found to be strongly related to W/C, RA replacement rate, as well as the parent concrete [9,42]. Curing conditions also have an impact on the mechanical properties of RAC, especially its tension resistance [43]. Usually, increasing the replacement rate of RA harms the strength of concrete, while adopting a higher curing temperature can alleviate the negative impact caused by RA [27]. With a well-designed W/C, concrete produced with mixed FRA and CRA can achieve comparable compressive strength and durability to NAC, but the splitting tensile strength and modulus of elasticity were found to be less affected [44]. As the strength of the parent concrete increases, the density, bond strength, and specific gravity of RCA as well as the resistance of RCA to abrasion, impact, and crushing all increase [35], thereby leading to improved concrete strength [22]. That is attributed to the higher strength and lower porosity of the attached mortar for RCA produced from a stronger parent concrete [35]. Some studies also investigated RA and RP produced from the concrete that had been recycled multiple times. It was found that RP produced from the parent concrete with more recycling cycles decreases the strength of concrete [25], but the recycling cycles of RA seem to have no obvious impact on some strength properties, such as the shear strength with confining stress [45]. It should be noted that RA sourced from the parent concrete that has already been affected by the alkali–silica reaction (ASR) significantly increases the risk of ASR in RAC, as the alkali released by RA can intensify ASR, causing the expansion of RAC [46]. Even if the alkali content is low in RA, ASR can still be triggered by the alkali from the cement matrix of RAC with the reactive particles provided by RA [46]. Thus, the parent concrete should be carefully selected to ensure the quality of RA produced. In addition to the condition of the parent concrete, the moisture state of RA was found to have a significant impact on the mechanical properties of RAC as well. RAC prepared with pre-saturated RA showed the highest compressive strength, followed by that prepared with oven-dried RA and saturated surface-dried RA [32]. That is because pre-wetting RA can enhance the internal curing effect driven by the self-drying of the cement paste, which promotes the secondary hydration of the cement paste through the release of water absorbed in RA [47]. Among all the mechanical properties of RAC, compressive strength was found to be the most sensitive to the changes in the moisture state, while modulus of elasticity and flexural strength were less affected by the moisture state [32].

Apart from the strength properties, RA also changes the behavioral response of concrete. Studies [24] found that RAC usually exhibits a more vertical failure plane under axial compressive loading. The ascending stage of the averaged axial compression stress–strain curve is less affected by RA, but the descending stage is much steeper when RA is incorporated. RA can reduce the modulus of elasticity, peak stress, strain at peak stress, and toughness of concrete, and finer RA tends to bring more uncertainty to these properties and the stress–strain behavior. Compared with NAC, RAC tends to have more irregular tensile failure and shows different patterns in the damage of triaxially confined regions and the asymmetric distribution of cracking locations in terms of compressive failure [48]. When subjected to dynamic compression [49], the replacement rate of CRA has little impact on concrete toughness but decreases the dynamic compressive strength. However, such a decrease in dynamic compressive strength is inhibited at a high strain rate. As the strain rate increases, the damage in RAC is exacerbated, while the dynamic compressive strength, toughness, and critical strain all increase. Similarly, RA can amplify the strain rate effect of concrete under splitting tensile loads as well, that is, higher strain rates cause an increase in the splitting tensile strength and stiffness [50]. Under monotonic and cyclic triaxial compression with confining pressure [51], three failure modes, including compressive failure, shear failure, and squeeze flow, can be observed in RAC. Both the replacement ratio of CRA and confining pressure can affect the strength properties and triaxial stress–strain behavior of RAC, with the confining pressure showing a more prominent impact. Higher confining pressure increases the loading stiffness while alleviating the post-peak softening of RAC.

Both the strength properties and the behavioral response of RAC are affected by, but not limited to, the microstructure of RA and ITZs. In addition, they are affected by RA geometry and the interaction between different RA particles. Crack development and mechanical behavior in RAC are directly related to the geometry of the attached mortar on RA [48,52]. The coverage of the attached mortar mainly affects the ultimate strength of RAC, while the amount of the attached mortar mainly affects the strain in the stress–strain response. Besides, the tips in RA also affect the mechanical properties of concrete negatively, particularly the compressive and shear properties, while the splitting tensile properties are less affected [53]. Such negative impact is governed by the number and orientation of tips as well as the elastic modulus of the aggregate. The tips aligned with the shear stress direction have minimal impact on concrete shear performance, while those perpendicular to the shear stress direction have the largest influence due to the proneness to strain concentration. RA, particularly CRA, can reduce the aggregate interlocking effect of concrete as well as the initial fracture toughness, unstable fracture toughness, and fracture energy [54]. A random arrangement of RA was found to be conducive to the strength of RAC compared with the array arrangement [52].

RAs from different sources has slightly different effects. For instance, compared with cement RCA, geopolymer RCA tends to cause a more obvious decrease in compressive strength but less decrease in tensile and flexural strength as well as the static modulus of elasticity [17], and RBA is more suitable for the production of lightweight concrete [31] due to its even lower density [55]. For RBA, a study on vegetative concrete showed a more significant impact of the replacement rate on compressive strength than W/C [56]. However, generally, RAs from different sources share similar properties and effects. Concrete incorporating RBA has similar compressive stress–strain curves to other RACs [57], and in some cases, RBA and RCA are even used together, with the optimal RBA-to-RCA ratio recommended as 5:5 [58]. Regardless of the sources of CRA, the coarse aggregate-to-cement ratio, the effective W/C, the replacement rate of CRA, as well as the water absorption of the coarse aggregate have the most significant impact on the elastic modulus of RAC, especially the water absorption, and a high value in either one of these four variables negatively affects the elastic modulus of RAC [59].

### 2.3. Durability

Research on the durability of RAC mainly focuses on the environmental conditions of salt attack, carbonation, wet–dry cycles, as well as freeze–thaw cycles.

(1) Salt attacks

Salt attacks faced by concrete mainly come from chloride ions and sulfates, particularly chloride ions, and account for 40% of the failures of concrete structures worldwide [60]. The higher porosity of RA than NA leads to a different chloride transport behavior in RAC compared with NAC [61]. Usually, RA can reduce the resistance of concrete to chloride attacks [20], but some literature also reported strengthening caused by chloride ions, as chloride ions can sometimes promote the formation of Friedel salt and C–S–H gel with a high Ca/Si ratio, thus lowering the porosity of RAC [62]. For NAC and RAC with a similar compressive strength, RAC was found to have comparable or even stronger resistance to chloride penetration [63]. Nonetheless, such improvement seems to be limited only to the early age of RAC with small amounts of Friedel salt and accelerated cement hydration. As the age of concrete increases, excessive Friedel salt, on the contrary, harms the performance of ITZs and causes later degradation in concrete strength [64]. Kang et al. [65] proposed that the transport of chloride ions in RAC is primarily governed by the diffusion coefficient as well as the volume and distribution of ITZs. An increase in the diffusion coefficient of ITZs causes an increase in the effective diffusion coefficient of concrete as well, but this effect is limited due to the relatively small volume of ITZs. However, since ITZs in RAC usually have a larger width, they can easily connect with cracks in the concrete to form ITZ–crack channels, which promote the transport of chloride ions. The volume fraction of RA is recommended to be kept below 35%, as within this range, the effect of RA on chloride transport is less obvious. The uneven distribution of RA in concrete significantly increases the effective diffusion coefficient of chloride ions. As for sulfate erosion [66,67], RAC tends to have more ettringite and gypsum generated under sulfate attacks compared with NAC, resulting in surface cracking and spalling of RAC as well as increased mass and brittleness and reduced relative dynamic modulus of elasticity (RDME) and plastic deformation capacity. Sustained compressive stress was found to be able to inhibit the formation of ettringite and gypsum in RAC and reduce the overall porosity, thereby significantly mitigating the loss of compressive strength and modulus of elasticity induced by sulfate erosion. However, the effect of sustained compression on plastic deformation capacity is less obvious.

(2) Carbonation

Carbonation primarily affects the service life of reinforced RAC, as it dilutes the alkalinity of concrete by reacting with the Ca(OH)_2_ within the carbonation depth [68]. Once the carbonation depth develops to the rebar surface, the passivation that should have protected the rebar from corrosion is damaged due to the weakened ambient alkalinity [69]. The carbonation depth of RAC is mainly determined by the exposure time, the compressive strength of concrete, the CO_2_ concentration, and the cement content [70]. However, even within one sample of RAC, carbonation depths can vary significantly. This leads to the increased inhomogeneity of RAC by dividing the concrete into irregular carbonated zones and uncarbonated zones. However, carbonation does not always act negatively on RAC. Instead, it can improve the mechanical performance of plain RAC by filling the pores and inner microcracks with the generated CaCO_3_ [68]. In addition, it was found to be able to decrease the average ITZ width by around 20% [36]. As a result, carbonation has been designed as an enhancement strategy, as will be elaborated in Section 5.1.2.

(3) Freeze–thaw/wet–dry cycles

Both RA and high W/C were found to have an adverse impact on the freeze–thaw resistance of concrete [71]. It was found in [72] that increasing the replacement rate of RA elevates the boundary stress during freezing and the central stress during thawing. A high W/C in the attached mortar on RA leads to increased stress at the boundaries, exacerbating local damage in RAC, while a high W/C in the cement matrix increases the pore and crystallization pressure within RAC, resulting in a higher stress level. Expanding the range of aggregate gradation can cause higher pore pressure, crystallization pressure, and principal stress in RAC. The loose and porous attached mortar on RA as well as the wider and more porous ITZs between the attached mortar and the cement matrix facilitate the formation of an interconnected crack network in RAC, exacerbating its freeze–thaw resistance [73]. However, there are also some opposite findings. As reported in [74], the W/C of the attached mortar on RA was found to have little impact on the resistance of RAC to freeze–thaw cycles. High permeability is conducive to enhancing the freeze–thaw resistance of RAC, as low permeability can lead to an elevated liquid pressure that promotes a higher tensile stress level. Similarly, due to the high porosity of RA, incorporating an appropriate amount of RA into concrete can also improve the freeze–thaw resistance. A greater range of aggregate gradation amplifies the freeze–thaw effect, while increasing the volume fraction of the aggregate, on the contrary, improves the freeze–thaw resistance of RAC as a result of the changed temperature distribution. Freeze–thaw cycles can significantly alter the morphology of hydration products in RAC and cause more inner pores and microcracks, thereby leading to the degradation of mechanical properties and freeze–thaw resistance [71]. However, freeze–thaw cycles have little impact on the composition of hydration products. A larger temperature difference in freeze–thaw cycles leads to greater pore pressure, crystallization pressure, and primary stress and causes more serious damage in RAC [74]. Compared with freeze–thaw cycles, the impact of wet–dry cycles was relatively less frequently reported individually. In [66], it was found that as the number of dry–wet cycles increases, both axial compressive strength and modulus of elasticity of RAC decrease. This can be attributed to the increased inner cracks caused by the dissolution and leaching of hydration products during dry–wet cycles [75].

Though different environmental conditions lead to different corrosion processes, the same end is reached that finally brings the degradation of the durability of RAC. That is, the formation of inner cracks. For salt attacks, inner cracks are induced by the volume expansion brought by the excessive chemical products deposited from the reactions between corrosive salts and concrete, while for freeze–thaw cycles, cracks come from the accumulated damage caused by the fluctuations in stress levels generated by the volume changes of water in different phases. Inner cracks arising under dry–wet cycles are believed to originate from the dissolution and leaching of hydration products in the concrete. Carbonation is an exception, in which the durability loss is triggered by the decreased concrete pH. Different environmental conditions may act together as well to form a coupling corrosion effect. For instance, the interconnected pores formed during freeze–thaw or dry–wet cycles promote the diffusion and transport of CO_2_ and ions, thereby exacerbating carbonation and salt corrosion.

## 3. Test Techniques

The commonly used test techniques in the research on RAC are summarized in Table 2.

In addition to the test techniques mentioned above, nondestructive testing and real-time monitoring techniques have also been used to investigate the deformation and damage evolution of RAC under different loading conditions. One of the commonly used monitoring techniques is acoustic emission (AE) monitoring, which detects inner fracture activities by collecting the acoustic signals released by newly formed cracks [104]. With the signals collected, the positions and distributions of inner cracks can be located [26,50], and both waveform analysis (e.g., amplitude, peak frequency, etc.) [54] and parameter analysis (e.g., *b*-value/*Ib*-value, AE hits, ring counts, cumulative energy, signal intensity, RA-AF analysis, etc.) [26,50,54,104] can be conducted on the signals to identify the failure modes and plot the development process of inner cracks with time [26,50,104]. It was found that both FRA and CRA significantly increase AE activities, the proportion of tensile cracks, the high-frequency amplitude, as well as the peak frequency of concrete [54]. Concrete incorporating CRA has more AE activities than that incorporating FRA [26]. Normally, monitored crack activities suddenly increase before the peak load [104], and extensive AE activities and a sudden spike in cumulative AE energy usually suggest the failure of the concrete [26]. However, in some cases, such as splitting tensile loading with high strain rates, crack activities increase from the onset [50]. Another commonly used real-time monitoring technique is digital image correlation (DIC), which is achieved through the relevant calculations from two digital images of the concrete before and after deformation [53]. DIC can be used to monitor the deformation [53,105] and surface damage progression [54,105] of loaded RAC.

## 4. Modeling

### 4.1. Analytical Models

#### 4.1.1. Physical and Mechanical Models

(1) Shrinkage

Shrinkage models for RAC are mostly developed based on the existing models for NAC. In [29], a model was established based on the previously proposed B4 model (Equations (1) and (2)) to predict the autogenous and drying shrinkage strain limits of RAC, in which the calculation of the parameters in the original B4 model (i.e., *a*, *w*, and *c*) was modified considering different water absorption of RA, the real aggregate content, as well as the attached mortar on RA.

(1) $$\varepsilon_{auto-NAC}=0.13\varepsilon_{auto-cem}a^{-0.75}w^{-3.5}c^{4.25}$$

(2) $$\varepsilon_{dry-NAC}=14.93\varepsilon_{dry-cem}a^{-0.80}w^{1.10}c^{-0.19}\rho^{-0.11}$$

Another modified model proposed to predict the autogenous and drying shrinkage of RAC with time was based on the shrinkage models for NAC specified in the EN standard [28], which took RA-related factors into account, including the differences in fineness ($k_{f}$) and elastic ($k_{e}$) moduli between RA and NA, the additional water of RA ($k_{w}$), as well as the coupling effect of CRA and FRA ($k_{cou}$).

For autogenous shrinkage:

(3) $$\varepsilon_{auto-RAC}\left(t\right)=\varepsilon_{auto-RAC}\left(\infty \right)\times k_{c}\left[1-\exp\left(-0.2k_{b}t^{0.5}\right)\right]$$

(4) $$\varepsilon_{auto-RAC}\left(\infty \right)=2.5k_{e}k_{w}k_{cou}k_{f}k_{ea}\left(f_{cu}-10\right)\times 10^{-6}$$

For drying shrinkage:

(5) $$\varepsilon_{dry-RAC}\left(t\right)=k_{e}k_{w}k_{f}k_{wc}k_{ea}\varepsilon_{dry-NAC}\left(t\right)$$

Other variables such as the effective W/C ($k_{wc}$), the expansion agent ($k_{ea}$), and the effect of the RA replacement rate ($k_{b}$ and $k_{c}$) were considered as well.

**Figure 3:**
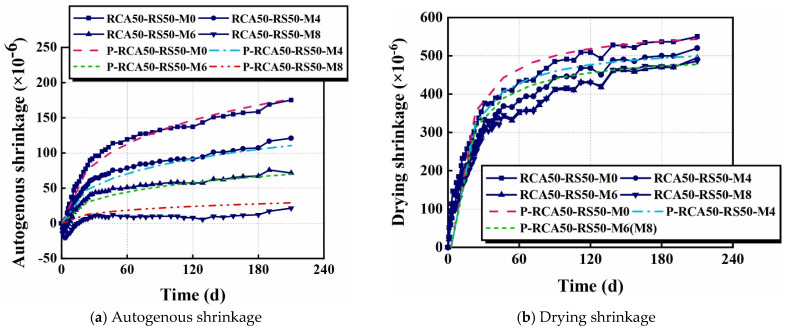
Validation of the modified EN shrinkage model [28].

The modified model has a higher accuracy in predicting autogenous shrinkage, while the drying shrinkage estimated by the model is slightly higher than the measured values (Figure 3).

(2) Strength and modulus of elasticity

Models have been proposed to predict the strength properties of RAC. Simply, formulae were proposed through regression analysis on the large amount of concrete data collected from the literature to predict the porosity, compressive strength, and elastic modulus of RAC based on the weighted water absorption of both natural and recycled coarse concrete aggregate as well as the effective W/C [106]. The early strength development of coarse recycled aggregate concrete (CRAC) was predicted using a logarithmic correlation with the maturity that can be calculated with either the Nurse–Saul method (applicable for temperatures between 0 and 40 °C) or the Hansen–Pedersen method [107]. Based on the Drucker–Prager criterion, multiscale models can be developed to predict the compressive strength of RAC considering the quasi-brittle failure mechanism [42].

As for the models for modulus of elasticity, they have been developed based on either (1) the elastic moduli of concrete prepared with pure NA ($E_{pure-NA}$) and pure RA ($E_{pure-RA}$) or (2) the basic parameters such as the replacement rate (r), coarse aggregate-to-cement ratio (a), water absorption (b), and effective W/C (c). The former strategy was adopted in [108] to develop the elastic modulus models based on effective medium approximation while considering the effect of Poisson’s ratio, as given in Equation (6), while the latter strategy was adopted by Kazmi et al. [59] to predict the elastic modulus of RAC with the replacement rate of CRA measured either by volume or weight, as specified in Equation (7) (by volume) and Equation (8) (by weight). The model given in Equation (6) seems to have better stability in terms of prediction accuracy.

(6) $$E=\frac{1}{4}\left[-\left(1-3r\right)E_{pure-RA}-\left(3r-2\right)E_{pure-NA}+\sqrt{{\left[\left(1-3r\right)E_{pure-RA}+\left(3r-2\right)E_{pure-NA}\right]}^{2}+8E_{pure-RA}E_{pure-NA}}\right]$$

(7) $$E=8-\frac{3-a}{-1.32+0.02ra-0.06r+0.45a}-\frac{a}{a{\left(e^{c}-b+5.3\right)}^{2}+c}-\frac{a+3b-48}{5c}+\exp\left(e^{c}\exp\left(-\frac{8}{\left(b-9.73\right)\left(r-11\right)}\right)\right)$$

(8) $$E=\frac{1}{8.21b-16.6-b^{2}}-1.8b+\exp\left(10.3c-2r-2a\right)-7\exp\left(5c-r-a\right)+\frac{10}{c}+\frac{1}{a\left(0.65-b\right)}+\frac{41.5}{46.4-ca^{4}}+12$$

(3) Axial compression

An improved stochastic model has been proposed to describe the stress–strain behavior of RAC under axial compression based on the fact that RA mainly affects the descending stage of the stress–strain curve while having little impact on the ascending stage [24]. In this model, the ascending stage of the stress–strain curve was still described with the existing model in Chinese standard GB 50010-2010 [109] (Equations (9) and (10)) with the shape factor *α* for this stage remaining unchanged, while for the descending stage, since the curve at this stage is usually much steeper in RAC, the shape factor for the descending stage adopted in the existing model (i.e., *β*) was modified accordingly considering RA-related factors, such as the replacement rate and the strength grade of the parent concrete from which the RA was produced:

(9) $$\sigma =\left(1-d\right)E\varepsilon$$

where the damage variable *d* is calculated as follows:

(10) $$d=\left\{\begin{array}{l} \begin{array}{cc} 1-\frac{{\alpha \sigma }_{peak}}{E\varepsilon_{peak}\left(\alpha -1+{\left(\frac{\varepsilon }{\varepsilon_{peak}}\right)}^{\alpha }\right)} & 0\leq \frac{\varepsilon }{\varepsilon_{peak}}<1 \end{array}\\ \begin{array}{cc} 1-\frac{\sigma_{peak}}{E\varepsilon_{peak}\left[\beta {\left(\frac{\varepsilon }{\varepsilon_{peak}}-1\right)}^{2}+\frac{\varepsilon }{\varepsilon_{peak}}\right]} & \frac{\varepsilon }{\varepsilon_{peak}}\geq 1 \end{array} \end{array}\right.$$

Compared with other models, the proposed model shows the highest accuracy in describing the stress-strain behavior of RAC under axial compression.

(4) Cyclic triaxial compression

A plastic damage constitutive model was established in [51] to describe the cyclic triaxial compression behavior of RAC, which was modified from the uniaxial plastic damage constitutive model of NAC integrating the two variables of confining pressure and the replacement rate of CRA, as the stress–strain curve of RAC under cyclic triaxial compression is quite akin to that of NAC under uniaxial compression. Specifically, the model was given as Equation (11), in which *d* is the damage variable, and parameters *m* and *n* were calculated based on their correlations with the confining pressure and the replacement rate of CRA obtained through fitting analysis:

(11) $$\frac{\sigma_{r}}{f_{cr}}=E_{0}\left(1-d\right)\left(\frac{\varepsilon_{r}}{\varepsilon_{cr}}-m{\left(\frac{\varepsilon_{u}}{\varepsilon_{cr}}\right)}^{n}\right)$$

**Figure 4:**
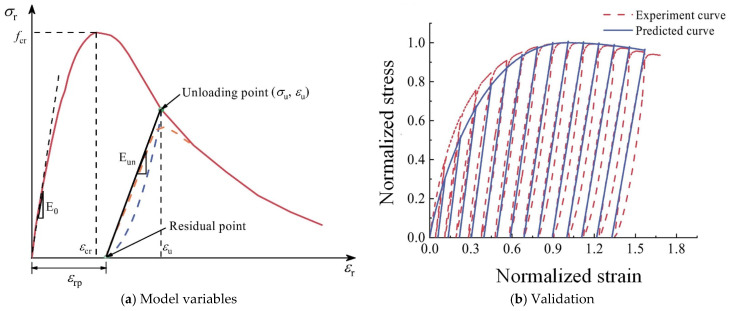
Cyclic triaxial compression model of RAC and its validation [51].

Different variables involved in the model are illustrated in Figure 4a, and the validation of the model is exhibited in Figure 4b. In general, the model has good accuracy.

(5) Water absorption

Simple prediction formulae were proposed in [98] through multiple linear regression analysis to calculate the sorptivity (*S*) and water saturation rate (*R_s_*) of RAC based on the electrical resistivity (*R*), percent volume of coarse RCA (*V*), as well as the air content of residual mortar (*A*), as given in Equations (12) and (13), respectively:

(12) S=25.449−4.669R−0.64V−0.074A+0.124RV

(13) $$R_{s}=311.192-55.049R-7.861V-0.912A+1.476RV$$

#### 4.1.2. Durability Models

(1) Carbonation

Mi et al. [36] presented a model to calculate the effective diffusion coefficient of CO_2_ in RAC ($D_{Effective}$) based on the coefficients of the cement matrix ($D_{CM}$) and ITZs ($D_{ITZ}$), as shown in Equation (14). The volume fractions of ITZs ($V_{ITZ}$) and the aggregate ($V_{A}$) were believed to have a significant impact on the diffusion of CO_2_, and the governing factors of stress ($f_{\sigma }$) and temperature ($f_{T}$) were included in the modeling as well:

(14) $$D_{Effective}=f_{\sigma }f_{T}\left[D_{ITZ}\frac{V_{ITZ}}{1-V_{A}}+D_{CM}\left(1-\frac{V_{ITZ}}{1-V_{A}}\right)\right]$$

(2) Chloride diffusion

An equivalent single-phase model [65] was proposed to describe the correlation between the diffusion coefficients of ITZs ($D_{ITZ}$), RAC ($D_{RAC}$), and the cement matrix ($D_{CM}$) considering the volume fraction of RA ($V_{agg}$) and the width ratio of ITZs to RA ($r_{w}$), as presented in Equation (15):

(15) $$\frac{D_{RAC}}{D_{CM}}=1+\frac{V_{agg}}{\frac{1}{2\left(\frac{D_{ITZ}}{D_{CM}}\right)r_{w}-1}+\frac{1-V_{agg}}{3}}$$

(3) Freeze–thaw/wet–dry cycles

Studies [71] suggested that the correlation between the mass loss rate of RAC and the number of freeze–thaw cycles can be described with a quadratic function, while that between the damage degree (damage variable) of RAC and the number of freeze–thaw cycles can be described with a power model. The axial compressive strength and peak strain of RAC as well as its stress–strain behavior when subjected to dry–wet cycles can be simply described through curve fitting [66].

### 4.2. Numerical Modeling

#### 4.2.1. Behavior Modeling

The finite element method (FEM) was commonly used in the numerical modeling of RAC to simulate either the mechanical behavior [52] or the environmental impact [65]. In these models, aggregates can be randomly generated as convex polygons or spherical shapes through the Monte Carlo method considering the requirements on aggregate gradation [72,74], while ITZs can be simply set to a fixed width without much accuracy loss [74]. It should be noted that the distribution of the attached mortar on RA will directly affect the simulation results. Studies [48] revealed that modeling the attached mortar at both ends of the larger radius of RA gives the most accurate prediction of the compressive and tensile behavior of RAC. Assuming the distribution of the attached mortar at both ends of the shorter radius of RA underestimates the mechanical response, while assuming the distribution as enclosing the aggregate overestimates the mechanical behavior.

#### 4.2.2. Prediction Models

In addition to the modeling of RAC behavior, prediction models based on machine learning algorithms have been attracting more and more attention in recent years. One of the most commonly used algorithms in prediction models is artificial neural networks [110], but it is actually not the one with the highest accuracy. Instead, tree-based algorithms such as the extreme gradient boosting and gradient boosting algorithms usually demonstrate better performance in predicting the strength properties of RAC such as elastic modulus and compressive strength [9,15,59]. Weighted methods can be integrated into these tree-based models to produce more efficient predictions [70]. For the purpose of strength prediction, four factors including the coarse aggregate-to-cement ratio, the effective W/C, the replacement rate of RCA (either by volume or by weight), and the water absorption of the coarse aggregate are suggested to be taken into account [59]. Apart from strength prediction, machine learning algorithms such as deep convolutional neural networks have also been used to estimate the mass and class of RA and classify different constituents simply based on 2D images, thereby speeding up the sorting and geometry characterization of RA in the recycling of C&D waste [111]. What should be emphasized is that it is necessary to balance the prediction accuracy and computation efficiency when selecting machine learning algorithms and consider such factors as the scale of the dataset and hyperparameters [110].

## 5. Modification and Improvement

**Figure 5:**
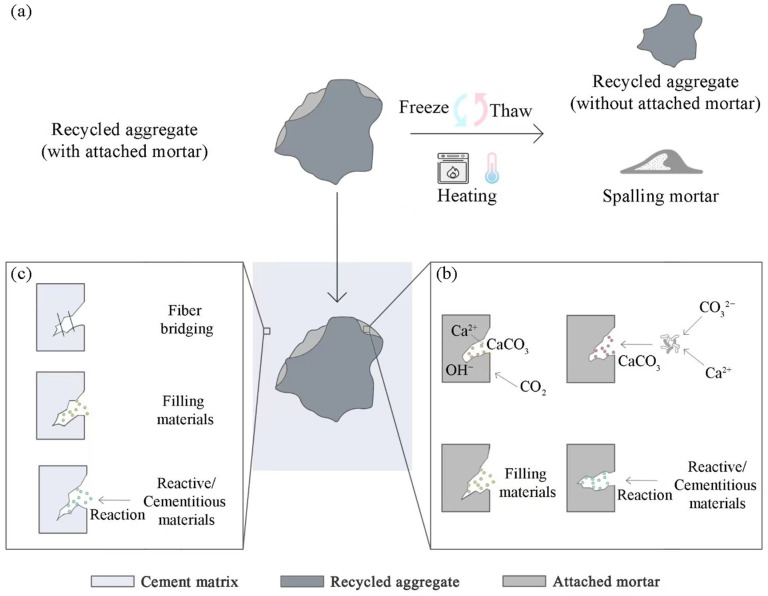
Modification and improvement of RCA and RAC. (**a**) Removal of the attached mortar; (**b**) Enhancement of the attached mortar; (**c**) Concrete improvement.

Modification of RCA mainly focuses on removing or strengthening the attached mortar, while the modification of RAC mainly acts on improving the microstructure [112], as depicted in Figure 5. Both paths aim to enhance ITZs in RAC and densify the concrete.

### 5.1. Aggregate Modification

#### 5.1.1. Removal of the Attached Mortar

The attached mortar can be removed from RCA through freeze–thaw cycle treatment [80] or heating at 600~700 °C [102,113], as shown in Figure 5a. The obtained RCA with the attached mortar removed can be directly used as CRA [102] or further crushed to form FRA [113]. Compared with untreated RCA, CRA obtained from heating shows a 67.7% lower water absorption, and the concrete produced with the treated CRA exhibits a 34% higher compressive strength as well as 22.9% and 20% lower porosity and capillary absorption, respectively, than those produced with untreated RCA [102]. The spalling mortar generated from the treatment can be used as either FRA [80] or heat-activated RP, and the latter, due to the high content of active C_2_S/CaO and inert quartz/calcite [102], promotes cement hydration and leads to a denser microstructure. It should be clarified that although the heating treatment can be energy-consuming, the issue could be alleviated through mass production with large equipment: only 5 tons of standard coal are required to treat 10,000 tons of RCA if large-scale rotary kilns are adopted, well below the level stipulated in the current environmental standards [113]. Furthermore, the heating treatment of RA could also be integrated into other high-temperature industrial processes such as steelmaking, which has been proven to be economically competitive and, if powered by green electricity, can even achieve zero emissions [114].

#### 5.1.2. Enhancement of the Attached Mortar

(1) Carbonation

**Figure 6:**
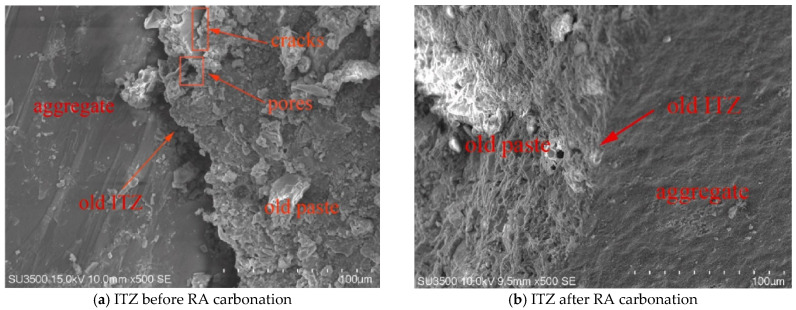
ITZ improvement in the recycled aggregate before and after carbonation [97].

Carbonation, as illustrated in Figure 5b, can enhance the old paste and ITZs around RA through the reaction between CO_2_ and the attached mortar on RA surfaces [97], which causes more CaCO_3_ (mostly calcite) to be precipitated to fill the pores while improving the elastic modulus of RA [34,53] and thereby enhances the strength properties and reduces the porosity, permeability, and water absorption of RAC [53,97]. Concrete produced with carbonated CRA can achieve a higher elastic modulus as well as compressive, splitting tensile, shear, and flexural strength compared with that produced with untreated RCA, and due to the Stefan effect, the former also has a higher dynamic peak stress while lower strain rate sensitivity of elastic modulus than the latter [14,53]. The synergistic effects of physical interlocking and chemical bonding can not only densify the surface of carbonated RCA, but also enhance the adhesion between CRA and the cement matrix in recycled concrete, thereby effectively improving ITZs [14]. The SEM images exhibited in Figure 6 intuitively show the improvement at ITZs. For RAC prepared with uncarbonated RA, microcracks mainly occur in old ITZs, while for that prepared with carbonated RA, microcracks tend to appear in new ITZs [53]. Carbonation of RA was found to have no obvious impact on the width of ITZs [14]. The compressive strength of recycled concrete follows a linear correlation with the average indentation modulus of ITZs between CRA and the attached mortar on it [14], and the evolution of splitting tensile damage is also affected by the elastic modulus and ITZ strength of the aggregate [53].

Similar to the carbonation of CRA, FRA can also be carbonated to reduce porosity and water absorption. Besides, RP after carbonation treatment was found to be able to facilitate the nucleation and stabilization of C–S–H gels during cement hydration and speed up the hydration due to its high pozzolanic reactivity [14]. As for carbonation techniques, CRA is more suitable for gas–solid carbonation, while RP is believed to be more suitable for liquid–solid carbonation [14].

To enhance the carbonation process, Luo et al. [82] proposed soaking CRA in calcium hydroxide before subjecting it to carbonation, so that more CaCO_3_ particles could be generated than after carbonation treatment alone to fill the pores and cracks in the attached mortar on CRA and thereby enhance its mechanical performance. It was found that the combined treatment of calcium hydroxide and carbonation can reduce the water absorption and the crushing index of CRA and increase the average indentation modulus of the interface between RA and the attached mortar. As a result, the compressive strength, splitting tensile strength, initial cracking load, peak load, Young’s modulus, initial toughness, unstable toughness, and the fracture energy of CRAC prepared with such modified CRA were significantly improved by 12.09%, 13.48%, 67.35%, 2.83%, 12.19%, 60.77%, 2.31%, and 5.38%, respectively, while the critical crack mouth and tip opening displacement were reduced by 9.13% and 10.96%. In comparison, only some of the mechanical properties, such as the initial toughness, the unstable toughness, and the fracture energy, witnessed an increase in the CRAC prepared with the RCA treated with single carbonation, while other properties, including the Young’s modulus, the critical crack mouth, and the tip opening displacement, all showed deterioration. Additionally, some studies combined the carbonation with recycled binder paste treatment [100], so that both the small binder paste particles and the calcite precipitated during carbonation fill the pores and voids in CRCA, thereby leading to lower porosity and water absorption of concrete as well as improved bonding of CRCA with the cement matrix.

(2) Sodium silicate treatment

Na_2_SiO_3_ solution treatment of RCA can improve the strength and durability of RAC, as Na_2_SiO_3_ in RCA can react with the weak C–H in cement hydration products to form stronger C–S–H, which fills the harmful pores and microcracks while strengthening ITZs in RAC and boosts the bond between RA and the cement matrix, thereby reducing the porosity and improving the compressive, flexural, and splitting tensile strength as well as the resistance of RAC to sulfate and acid attacks [58,92]. In some studies, nano-SiO_2_ has been used together with Na_2_SO_3_ treatment to further enhance the properties of the RA and the RAC produced [15].

(3) Coating

Coating RA with either filling or reactive materials is also effective in enhancing its properties (Figure 5b). As for the filling materials such as crushed RCA powder, they can fill the pores in the cement matrix and the attached mortar on RA, thereby improving the physical and mechanical properties of RAC [101]. As for reactive materials, in addition to the sodium silicate mentioned above, RP can be used to coat RA surfaces as well. CRA wrapped by RP can improve the fluidity and compressive strength (by 3.66%) of RAC, as the concrete powder attached to CRA surfaces facilitates extensive pozzolanic reactions, thereby leading to a denser microstructure and higher strength and microhardness of ITZs [78]. Siletani et al. [89] reported silica fume, nano-silica, micro-zeolite, and nano-montmorillonite as coating materials for RA modification. It was found that RA treated with silica fume had the best performance in improving the density, mechanical properties, and durability of concrete, while that treated with micro-zeolite achieved the best improvement specifically in compressive strength and the resistance to chloride ion penetration. RA modified by these pozzolanic materials had a smaller width and fewer microcracks and pores in new ITZs (i.e., the interface between the cement matrix and the attached mortar on RA) when used to prepare RAC, but it had less impact on old ITZs. Portland cement and aluminous cement can also serve as reactive coating materials [115], but the molar ratio of Al_2_O_3_ to Fe_2_O_3_ as well as the cooling conditions following calcination are recommended to be specially controlled, so that more sufficient hydration could happen to effectively fill the pores and promote strength development once the coated RA is used in concrete mixing [116]. In addition to these filling or reactive powders, polymers such as epoxy resin were also used to wrap RA, which can reduce microcracks in RAC and narrow ITZs while improving their strength through strong bonding [81].

(4) MICP treatment

Microbially induced carbonate precipitation (MICP) treatment acts similarly to carbonation, that is, filling pores and microcracks in RA with precipitated CaCO_3_ (Figure 5b). MICP-treated RA generally demonstrates decreased water absorption and increased apparent density [94]. Concrete produced with MICP-treated RA can achieve an 11% higher splitting tensile strength than NAC and comparable flexural and compressive strength, and similar failure patterns and stress–strain behavior to NAC can be observed under uniaxial compression [94,105]. Some measures have been taken to enhance the effect of MICP. For example, mesoporous silica nanoparticles were used as the carriers for urea to mediate the precipitation kinetics of CaCO_3_ in the MICP treatment of RCA, which significantly increased the cohesion of the precipitated CaCO_3_ by 25% and the adhesion of CaCO_3_ to RCA by 37% [117]. To address the issue that CaCO_3_ can hardly be precipitated uniformly due to the uneven distribution of bacteria, Luo et al. [88] proposed pretreating RA with chitosan solutions before subjecting to to MICP treatment, so that CaCO_3_ (mainly calcite in phase) can be more uniformly and densely precipitated both on the surfaces and in the pores of CRA. A 2.5% increase in apparent density as well as 20.6% and 18.1% decrease in water absorption and the crushing index were observed, respectively, in the CRA with combined treatment of chitosan solutions and MICP, which, when used in concrete, could bring an improvement in the workability, the compressive strength, as well as the chloride penetration resistance of the concrete and reduce the capillary water absorption. In [86], sodium alginate was used to improve the uniformity of precipitated CaCO_3_ on RA surfaces treated with MICP through the uniform distribution of bacteria on RA surfaces achieved by sodium alginate and the network structure of Ca–alginate. Such treatment was found to be able to effectively improve the cohesion of precipitated CaCO_3_ as well as the bonding strength between CaCO_3_ and RA and reduce the water absorption and the crushing index of RA. Concrete prepared with treated RA exhibits a 15.3% lower saturated water absorption. In recent years, studies on the phase composition of concrete under the effect of bacteria have revealed the potential to expand the functionality of MICP, in which diverse mineral phases other than CaCO_3_, such as thaumasite and pseudo-crystalline C–S–H, were precipitated to form a compact microstructure. This can be achieved simply through the treatment with the waste slurry from the industrial fattening of livestock [118], which not only enhances the performance of RAC but also finds a way to ensure efficient utilization of agricultural wastes, thereby further fostering the sustainability of concrete.

Even though various techniques have been developed to improve the treatment effect of MICP, it still faces many challenges, such as the temperature and pH values required by bacteria to maintain activity, which restrict its large-scale application. The same predicament also applies to its variant, enzyme-induced carbonate precipitation (EICP), which uses extracted enzymes instead of bacteria to mediate carbonate precipitation [119]. In response, some researchers started to try to induce CaCO_3_ precipitation without bacteria. For instance, in one study [87], RA was first soaked into the mixed solution of urea and calcium acetate, which was then subjected to water bath heating so that the urea decomposed to release CO_3_^2−^ that could react with Ca^2+^ to form aragonite in the pores and microcracks of RA. Such treatment significantly reduces the water absorption and the crushing index of RA and increased the slump, compressive strength, and splitting tensile strength while decreasing the capillary water absorption and the electrical flux (i.e., the enhanced resistance to chloride ion penetration) of RAC when the treated RA was used to replace regular untreated RA. In recent years, biomimetic carbonate precipitation has been explored [120], which utilizes such molecules as L-Asp [121,122,123] to induce stable CaCO_3_ precipitation with superior strength properties. This has exhibited great potential in RA enhancement.

### 5.2. Concrete Improvement

(1) Supplementary cementitious materials

As depicted in Figure 5c, supplementary cementitious materials play a role as both the filling materials and the reactive materials in RAC improvement. The normally used supplementary cementitious materials include ground granulated blast furnace slag [50,95], fly/bottom ash [34,124], and micro/nano-silica or silica fume [99]. Metakaolin is sometimes incorporated into RAC as well to reduce the void volume and the water absorption [15]. It was found that silica fume can reduce the workability of concrete due to its large specific area while improving the compressive strength, tensile strength, flexure strength, and elastic modulus through pore filling and pozzolanic effects [91]. In addition, silica fume can also reduce the water absorption and enhance the resistance of concrete to acid attacks [91]. In RAC produced with slag and fly ash, increasing the slag content can promote the formation of a calcium–silicate gel, thereby improving the strength of CRAC, reducing the porosity and the mass loss induced by water evaporation, and alleviating the drying shrinkage and crack generation [27]. Such RAC with slag and fly ash has significantly lower electrical resistivity value, rapid chloride penetration test value, and sorptivity, indicating lower water permeability and better resistance to chloride and sulfate attacks [90]. However, it is also more vulnerable to carbonation due to reduced alkalinity [90]. When exposed to elevated temperatures [77], the compressive strength of RAC with slag and fly ash first increases with the increase in temperature, reaches the peak at around 200 °C, and then decreases sharply when the temperature exceeds 600 °C. At temperatures below 400 °C, more C–A–S–H and high-calcium C–(N)–A–S–H gels are generated in O–N-type ITZs due to the migration of Ca^2+^ from the attached old mortar, making them denser than the A–N-type ITZs. At temperatures over 400 °C, porosity increases in both ITZs and the cement matrix due to the decomposition of gels, and cracks between the aggregates and ITZs expand significantly. All this leads to the degradation of RAC under high temperature. Slag was found to be able to enhance the residual strength of RAC after its exposure to high temperature. A metakaolin amount below 15 wt% of the binder can improve the mechanical properties (particularly compressive strength) and carbonation resistance of fly ash-incorporated RAC, as active SiO_2_ and Al_2_O_3_ in metakaolin can react with Ca(OH)_2_ to generate C–S–H gels, which fill the pores and reduce the alkaline content [33]. Together with fly ash, this improves the pore size distribution in RAC.

(2) Fiber reinforcement

Fibers can inhibit the crack propagation and improve the toughness of RAC [26] through the bridging effect (see Figure 5c). Research has been conducted on various types of fibers to determine their enhancement effect. Steel fibers [104] and glass fibers [125] can improve the bending performance of RAC. Flax fibers [49] are effective in reducing the damage while increasing the critical strain of RAC under dynamic compression. A suitable volume of polypropylene fibers can significantly improve the axial compressive strength, the residual tensile strength, the modulus of elasticity, and the durability of RAC, while excessive use of polypropylene fibers causes a negative effect [79,85]. Carbon fibers [91] can reduce the workability of concrete due to its high water absorption while improving the compressive strength, tensile strength, flexure strength, and elastic modulus through crack-bridging effects. In addition, carbon fibers can also enhance the resistance of concrete to acid attacks. However, carbon fibers increase the porosity and water absorption of concrete as well. Silica fume was found to be able to improve the bonding between the cement matrix and carbon fibers and promote a good distribution of carbon fibers in concrete. It is worth noting that fibers used for RAC reinforcement can be recycled from waste to further enhance sustainability. For example, glass fibers recycled from waste wind turbines have been used as additives in Portland and aluminate cement to improve mechanical properties and durability [126].

## 6. Application

Restricted by inferior performance and other hindering factors such as the underdevelopment of the supply chain as well as the lack of incentives and supervision in C&D waste recycling [127], RA and RAC are currently still not applied in real engineering on a large scale. However, some preparations have been made for their application. In terms of concrete design, the water reduction method has been proposed for the mix design of RAC [84]. It is effective for designing RAC containing both CRA and FRA, even that containing PP, which calculates the natural coarse and fine aggregate and increases the coarse-to-fine aggregate ratio by volume while reducing the W/C according to the attached mortar on CRA and the cement paste content of FRA. The water reduction method can improve the strength while reducing the slump, shrinkage, and creep of RAC produced purely with FRA or a mix of CRA, FRA, and RP. Another method was developed for the design of self-compacting RAC with recycled coarse and fine aggregate, cement, as well as fly ash, in which the content of NA and RA was first determined based on the target slump flow, and the effective W/C was then calculated based on the target compressive strength [103]. In addition to the procedure optimization, machine learning was also introduced into concrete design to intelligently generate the mix design that could meet the target compressive strength [9]. To make reinforced RAC, the bond performance and the bond failure mode of the rebar embedded in RAC were tested, based on which bond–slip models were proposed [128].

Structural components manufactured from RAC have been intensively explored, mostly concrete-filled steel tube/steel-confined components [129,130,131,132,133,134] or components enhanced with a carbon fiber-reinforced polymer [135,136]. Apart from mechanical behavior, these components were also tested in special conditions, such as fire exposure. For components subjected to high-temperature conditions, the component temperature was found to decrease with the increasing content of CRA or FRA [137]. The temperature distribution in RAC components was observed to be similar to that in components made of regular concrete, and models have been proposed to predict the inner temperature of one-dimensional (e.g., slabs or walls) and two-dimensional (e.g., columns or beams) RAC components under high temperature [137].

As for RA and RAC in real engineering, there is a consensus that they are suitable for the construction of pavements, particularly those in pedestrian or light traffic areas [76,93,96,138,139,140]. More functions of RAC have been developed, such as pavements that can eliminate the atmospheric pollutants released by vehicles [141] or pervious concrete that can be used for water purification [142], in which photocatalysts such as TiO_2_ are integrated into RAC to form photocatalytic construction materials [143]. In the vacuum preloading of dredged sludge [144], recycled fine concrete aggregate was used as the horizontal drainage cushion to alleviate the clogging of the prefabricated horizontal drain [145]. All these have revealed wider scenarios in the future application of RA and RAC.

Application research on RA and RAC is summarized in Table 3.

## 7. Conclusions and Recommendation

In this paper, recent advances in RA and RAC research were systematically reviewed, including their properties, commonly used test techniques, material modeling, the modification and improvement methods, as well as their engineering applications. Conclusions are summarized as follows:

(1) Compared with NA, RA usually has lower density and higher water absorption. In fresh concrete, RA can reduce the workability, increase the water consumption during concrete mixing, and shorten the setting time of fresh concrete. In hardened concrete, RAC usually exhibits higher porosity and air content, increased water and capillary absorption, as well as lower thermal conductivity. RA increases the drying shrinkage while alleviating autogenous shrinkage, which is affected by its size and the strength of the parent concrete. Adopting a two-stage mixing approach can have a positive effect on the workability and drying shrinkage of RAC.

(2) RA was generally found to act negatively on the mechanical properties of concrete, leading to decreased strength and modulus. That is attributed to the porous microstructure of the attached mortar and weaker ITZs formed in RAC. However, opposite results were also reported, showing that RA may also contribute positively to concrete strength, which has been explained from the perspective of water absorption and secondary pozzolanic reactions. The strength properties and the failure mechanism of RAC are strongly related to the W/C, the RA replacement rate, the curing conditions, as well as the parent concrete. RA geometry and distribution also have an impact. Different failure modes and mechanical responses from NAC have been observed in RAC, but they also share some similarities in behavior, such as the ascending stage in compression stress–strain curves.

(3) RA usually reduces the resistance of concrete to salt attacks and freeze–thaw cycles. However, different results have also been reported, suggesting that RA may, on the contrary, enhance the freeze–thaw resistance of concrete and witness an increase in concrete strength in chloride-rich environments. Dry–wet cycles are harmful to both the axial compressive strength and the modulus of elasticity of RAC, and carbonation reduces the average ITZ width while increasing the inhomogeneity of RAC by dividing the concrete into irregular carbonated zones and uncarbonated zones.

(4) Techniques commonly involved in RAC research include mechanical tests, durability tests, and physical property tests (macroscale), pore structure analysis (mesoscale), as well as characterization methods (microscale). Real-time monitoring techniques have also been used to monitor the fracture process and deformation of RAC under loading, such as AE and DIC.

(5) Both analytical and numerical models have been developed to describe or predict the behavior or properties of RAC. For analytical models, they are usually modified from the existing models for NAC or established through regression analysis. For numerical models, random methods such as Monte Carlo have always been used to generate RA, and the distribution of the attached mortar should be taken into account to improve accuracy. Machine learning methods have been widely tested and applied in predicting the properties of RAC.

(6) Modification of RA can be implemented by either removing or strengthening the attached mortar, while the modification of RAC is mainly achieved by improving its microstructure. The attached mortar on RA can be removed by freeze–thaw treatment or heating, or enhanced through carbonation, sodium silicate treatment, coating, or MICP treatment. RAC can be reinforced with fibers or improved using supplementary cementitious materials.

(7) Current research on RAC application mainly focuses on the optimization of the concrete mix design and procedures, structural components such as concrete-filled steel tube/steel-confined components or components enhanced with a carbon fiber-reinforced polymer, as well as pavement and multifunctional construction materials.

For the sake of engineering, the following recommendations are made:

(1) In terms of raw materials, RA is recommended to be produced from the parent concrete with a higher strength and fewer recycling cycles, and the parent concrete should not be affected by ASR. RA with smaller sizes, lower water absorption, narrower range of gradation, fewer tips and alkali-reactive particles, as well as higher contents of silica, alumina, and calcium is favorable. Pre-saturating RA before use is helpful in improving the compressive strength of concrete. If necessary, measures can be taken to remove or enhance the attached mortar on RA surfaces.

(2) In terms of concrete preparation, lower coarse aggregate-to-cement ratio, replacement rate, and effective W/C are suggested, and RA should be evenly but randomly distributed in concrete. Adopting a two-stage mixing approach and a higher curing temperature are beneficial. Adding supplementary cementitious materials or fibers into concrete can alleviate the strength loss brought by RA.

(3) In terms of design and construction, analytical and numerical models developed for NAC can still lead to reliable results if RA-related factors are incorporated, and machine learning methods can be used to power the design. To improve the structural performance, steel- or fiber-reinforced polymers can be employed to apply confinement to RAC, and multiscale techniques can be deployed to guarantee and monitor structural health. Based on the special properties of RA and RAC, broader application scenarios can be exploited.

## Figures and Tables

**Table 1:** Impact of recycled aggregate on the basic physical properties of concrete.

Properties	Impact	Properties	Impact
Density	−	Setting time	−
Void volume/porosity	+	Segregation resistance	−
Air content	+	Drying shrinkage	+
Water/capillary absorption	+	Autogenous shrinkage	−
Workability/slump	−	Thermal conductivity	−

Note: + represents increment, −—decrement.

**Table 2:** Test techniques for recycled aggregate concrete.

Scale	Category	Test Techniques
Macroscale	Mechanical tests	For concrete: Compressive test [38,76,77,78,79,80], splitting tensile test [29,43,81], flexural/bending test [17,82], elastic modulus test [27,44], Young’s modulus test [32], shear test [53], monotonic/cyclic triaxial test [51,83], relative dynamic modulus of elasticity test [66,71], split Hopkinson pressure bar test [49], creep test [84], residual flexural tensile strength test [85], ultrasonic pulse attack [86], etc. For aggregate: Los Angeles abrasion test [35,43], crushing value test [58,82,86,87,88], impact test [35], etc.
	Durability tests	Carbonation/accelerated carbonation test [33,36], electrical resistivity test [89,90], (rapid) chloride penetration test [63,89], sulfate erosion test [66], acid attack test [91,92], dry–wet cycle test [61,93], freeze–thaw cycle test [71,94], half-cell potential test [95], corrosion rate test [95], electric flux test [87], etc.
	Physical property tests	For aggregate or hardened concrete: Water absorption and capillary absorption test [25,88], permeability test [96], hardened density test [97], sorptivity test [90,98], water reversion test [23], ultrasonic pulse velocity test [31,99], thermal conductivity test [18,100], thermal diffusivity test [101], autogenous and drying shrinkage test [28,34], air content test [71], mercury intrusion porosimetry test [22,62], vacuum saturation test [102], specific gravity test [35], etc. For fresh concrete: Slump test [24,103], flow table test [25], compacting factor/drop test [15,19], Kelly ball penetration test [15], consistency test [21], setting time test [21], J-ring test [26], V-funnel test [26], rheometer test [26], etc.
Mesoscale	Pore structure analysis	X-ray computed tomography (X-CT) [23,38,73], nuclear magnetic resonance (NMR) [58], 3D surface topography [93], etc.
Microscale	Characterization methods	For morphology analysis: Scanning electron microscopy (SEM) [38,46,66,100], backscattered scanning electron microscopy (BSEM) [36,37,76], etc. For chemical composition: X-ray diffraction (XRD) [38,61,76], thermogravimetric (TG) analysis or thermal gravimetry/derivative thermal gravimetry (TG/DTG) [38,61,96], energy-dispersive spectrometry (EDS) [46,76,93], X-ray fraction (XRF) [23,90], Fourier-transform infrared (FTIR) spectroscopy spectrum [22,30], etc. For micromechanics: Nanoindentation test (NIT) [38,92,93], microhardness test [78], etc.

**Table 3:** Current research on the application of recycled aggregate and recycled aggregate concrete.

Branch	Topics	Cases
Concrete design	Mix optimization	Water reduction method
		Target workability and strength-based mix design
		Machine learning design method
	Reinforced RAC	Rebar behavior in RAC
Components	Composite structure	RAC-filled steel tube/steel-confined components
		Carbon fiber-reinforced polymer-confined RAC
Real engineering	Pavement engineering	Photocatalytic pavement
	Geotechnical engineering	Drainage of foundations

## Data Availability

No new data were created or analyzed in this study. Data sharing is not applicable.

## Nomenclature

AE	Acoustic emission
ASR	Alkali–silica reaction
BSEM	Backscattered scanning electron microscopy
C&D	Construction and demolition
CRA	Coarse recycled aggregate
CRAC	Coarse recycled aggregate concrete
DIC	Digital image correlation
DTG	Derivative thermal gravimetry
EDS	Energy-dispersive spectrometry
EICP	Enzyme-induced carbonate precipitation
FEM	Finite element method
FRA	Fine recycled aggregate
FRCA	Fine recycled concrete aggregate
FTIR	Fourier transform infrared
ITZ	Interfacial transition zone
MICP	Microbially induced carbonate precipitation
NA	Natural aggregate
NAC	Natural aggregate concrete
NIT	Nano-indentation test
NMR	Nuclear magnetic resonance
RA	Recycled aggregate
RAC	Recycled aggregate concrete
RBA	Recycled brick aggregate
RCA	Recycled concrete aggregate
RCP	Recycled concrete powder
RDME	Relative dynamic modulus of elasticity
RP	Recycled powder
SEM	Scanning electron microscopy
TG	Thermogravimetric
UPV	Ultrasonic pulse velocity
W/C	Water-to-cement ratio
X-CT	X-ray computed tomography
XRD	X-ray diffraction
XRF	X-ray fraction
